# Nursing informatics competency and its associated factors among palliative care nurses: an online survey in mainland China

**DOI:** 10.1186/s12912-024-01803-5

**Published:** 2024-03-05

**Authors:** Junchen Guo, Junqingzhao Liu, Chaoyi Liu, Ying Wang, Xianghua Xu, Yongyi Chen

**Affiliations:** 1 Department of Palliative Care, Hunan Cancer Hospital, No. 283, Tongzipo Road, Yuelu District, 410006 Changsha, Hunan China; 2 School of Nursing, University of South China, No. 28, Changsheng West Road, 421001 Hengyang, Hunan China

**Keywords:** Nursing informatics competency, Palliative care, Nurses, Cross-sectional study

## Abstract

**Background:**

Nursing informatics (NI) competency is a required core competency for high-quality care in digitally enabled healthcare environments. Given the increasing reliance on digital health in palliative care settings, it becomes crucial to evaluate the NI competency of nurses to ensure the seamless integration and effective utilization of digital health in their clinical practice. This study aimed to investigate the level of NI competency and explore its associated factors among palliative care nurses in mainland China.

**Methods:**

A cross-sectional design was conducted for this study, involving a total of 409 palliative care nurses from 302 hospitals in mainland China. Anonymous data were collected through a self-designed sociodemographic questionnaire, the Nursing Informatics Competency Scale (NICS) and the Innovative Self-Efficacy Scale.

**Results:**

The total score of the NICS was 129.19 ± 22.02, which indicated that Chinese palliative care nurses had a moderate level of NI competency. There was a positive correlation between innovative self-efficacy and NI competency (*r* = 0.602, *P* < 0.01). The hospital level and innovative self-efficacy were identified as statistically significant factors influencing nurses’ NI competency based on multiple linear regression analysis results. These associated factors could explain 35.1% of the difference in NI competency.

**Conclusions:**

This study found that palliative care nurses in mainland China exhibited moderate levels of NI competency and identified the hospital level and innovative self-efficacy as associated factors of nurses’ NI competency. Measures such as developing supported strategies, including targeted NI training programs by nursing education managers of primary-level hospitals and creating a positive culture of innovation by healthcare institutions can be considered to improve the level of NI competency among Chinese palliative care nurses.

**Supplementary Information:**

The online version contains supplementary material available at 10.1186/s12912-024-01803-5.

## Introduction

Palliative care is pivotal in improving the quality of life for patients facing life-limiting illnesses. It encompasses a wide range of supportive interventions that address physical and psychological, social, and spiritual needs [1]. With the increasing ageing population and the rise in non-communicable diseases, there is a rapidly growing global demand for palliative care services. According to the World Health Organization, an estimated 56.8 million people require palliative care annually, with China potentially contributing significantly to this number due to its population exceeding 1.4 billion [2, 3].

As the need for palliative care continues to surge, it is imperative to educate and train healthcare professionals specialized in this field, particularly palliative care nurses, who play a crucial role in delivering high-quality care to end-of-life patients and their families [4]. However, nurses face challenges when providing palliative care in mainland China, especially in home-based settings, predominantly due to the scarcity of palliative care nurses cannot meet the increased demand for home-based palliative care [5]. According to data from the Palliative Care Committee of the Chinese Nursing Association in 2022, specialized education and training in palliative care has been completed by only 1,132 nurses [6].

With the advancement of modern information technology, digital health (DH) emerges as a transformative healthcare service model with the inherent potential to elevate and extend palliative care services on a global scale [7]. Existing studies have highlighted the numerous benefits of DH, which encompass its remarkable ability to ensure seamless continuity of care and effectively address the ever-growing demands for palliative care services [8, 9]. Particularly in remote areas with limited resources, DH has emerged as a valuable solution to bridge the gap and improve access to quality care [10]. From 2022 to now, the National Health Commission of the People’s Republic of China has continued to emphasize the importance of innovation in nursing services and explicitly highlighting the utilization of DH to provide more convenient and efficient care to end-of-life patients [11]. As a result of these forward-looking policies and initiatives, there has been a swift and remarkable expansion of nurse-led DH-based palliative care services in a number of hospitals in recent years [12, 13].

Amid the ongoing digital transformation of palliative care services, one critical aspect that emerges is the concept of “Nursing Informatics (NI) competency”. NI competency refers to a satisfactory level of knowledge, skill and ability to successfully accomplish specific informatics tasks, and it is acknowledged as a fundamental proficiency for nurses [14]. According to International Medical Informatics Association, NI competency is a required core competency for high-quality care in digitally enabled healthcare environments and is consisted of three domains, including computer skills, informatics knowledge, and informatics skills [15]. The synergy among these domains is essential for nurses to achieve the NI competency. Computer skills are the foundational element for fostering a solid groundwork in NI competency, informatics knowledge provides the intellectual framework of NI competency, which involves a deeper understanding of the theoretical underpinnings of healthcare information systems, data management, and the integration of technology into clinical nursing practice, and informatics skills bring theory into practice, contributing significantly to the overall NI competency [15]. Several studies have examined the baseline levels and factors associated with NI competency among clinical nurses or nursing undergraduate students [16–19]. The results varied across studies conducted in different countries, for instance, research in Iran and Korea revealed below-average levels of overall NI competency among nurses [16–17], while studies in Canada indicated a moderate level in NI competency [18]. Beyond demographic factors such as age, educational qualifications, years of experience, job position and work setting [16–18], one essential factor related to NI competency is innovative self-efficacy, which refers to an individual’s confidence in their ability to use new technologies to perform their job proficiently [19, 20]. Nurses with higher levels of innovation self-efficacy exhibit a stronger inclination to address clinical nursing problems and actively acquire relevant resources and information [19]. They proactively explore novel working methodologies, models, and technologies to enhance their informatics competency.

Given the increasing reliance on DH in palliative care settings, it becomes equally crucial to evaluate the NI competency of palliative care nurses to ensure the seamless integration and effective utilization of DH in their clinical practice. However, research on the current status of NI competency and factors affecting NI competency in palliative care settings has yet to be investigated, especially in the context of mainland China. Compared to other countries with established comprehensive frameworks for palliative care services, China is in the process of integrating palliative care into its evolving healthcare system. However, the distribution of DH-based palliative care services resources, including facilities and trained professionals, may vary across regions [6]. Furthermore, the nursing education programs in mainland China do not adhere to standardized criteria for teaching NI at present, which may contribute to variations in the NI competency of Chinese palliative care nurses compared to their counterparts in other nations [21]. Given the differences in conditions between China and other nations, it is necessary to bridge this gap. Therefore, this study aims to (1) investigate the level of NI competency among palliative care nurses in mainland China, and (2) identify the factors associated with NI competency.

## Methods

### Study design

In this study, a cross-sectional design was implemented. The Strengthening the Reporting of Observational Studies in Epidemiology (STROBE) checklist was chosen to conduct and report.

### Participants and sample size

From July 2022 to August 2022, a total of 409 palliative care nurses from 302 hospitals or healthcare institutions across China were investigated using a convenient sampling method. The inclusion criteria for participants were as follows: (1) successfully obtained the Chinese palliative care nurse qualification certificate, (2) currently working in the palliative care unit, and (3) directly providing palliative care for patients. Nurses who refused to participate in this study were excluded. The sample size of this study was calculated based on the sample size calculation principle used in Kendall’s cross-sectional investigation: N = independent variable × (5~10) [22]. The independent variables included sociodemographic information, the Nursing Informatics Competency Scale score (5 dimensions) and the Innovative Self-Efficacy Scale score (single-dimension scale). Considering a potential 20% rate of invalid questionnaires, a range of 90 to 180 participants would be necessary to achieve sufficient data for analysis.

### Procedure

We employed an anonymous online survey conducted through Questionnaire Star, a tool designed for questionnaires, and distributed to some Chinese palliative care nurse online groups via WeChat to ensure participants had the flexibility to respond at their convenience. The survey began with the first page containing the participant informed consent form, where detailed information is provided regarding the objectives of the study, time commitment, potential risks, benefits of participation, and avenues for lodging complaints. Subsequently, participants were presented with a singular question, “Would you like to participate in this study?“, and were provided with response options of “Yes” or “No”. Opting for “Yes” indicated their voluntary consent to participate, and then they were directed to proceed with completing the questionnaire. Before the formal survey, two researchers who were very familiar with the research topic completed the questionnaire to ensure the collection of high-quality data. The minimum time they spent completing the survey was 120 s. A total of 415 questionnaires were returned, of which those that responded within a short period of time and with very regular answers, such as the whole questionnaire was answered with “agree”, were excluded. Finally, 409 were valid for analysis, giving an effective response rate of 98.55%.

### Measurements

#### Sociodemographic characteristics

The sociodemographic characteristics of the study were collected by a self-designed questionnaire, and we invited six palliative care experts and used a five-point Likert scale from 1 (not necessary) to 5 (very necessary) to evaluate the degree of importance of each sociodemographic variable. The content validity index was 0.956 based on the expert’s scores. The final version of the questionnaire included gender, age, marital status, educational background, hospital level, professional title, monthly income (RMB), employment category, and years of experience in palliative care. Following the guidelines stipulated in the “Chinese Hospital Classification Management Standard (2022 version),” the Chinese government classifies hospitals as three levels based on their size, infrastructure, and equipment provisions (Table 1) [23].

**Table 1:** The classification standard of Chinese hospitals

Hospital level	Description
Primary level	To provide preventive, medical, health care, and rehabilitation services directly to communities of a certain population.
Secondary level	To provide comprehensive medical and health services to multiple communities and undertake certain teaching and research tasks
Tertiary level	To provide comprehensive medical and health services to several regions, and hospitals with higher education and scientific research missions that are more than regional in scope.

#### Nursing informatics competency

Luo et al. developed the Nursing Informatics Competency Scale (NICS), a self-reporting scale designed to assess nurses’ NI competency [24]. The scale consists of 32 items and covers five domains: nursing informatics awareness, computer operational capability, computer management capability, nursing informatics operation skills, and nursing informatics management skills. The nursing informatics awareness refers to the extent to which individuals are familiar with the concepts, principles, and applications of nursing informatics. The computer operational capability reflects an individual’s proficiency in using computer systems and performing basic operations. The computer management capability encompasses the ability to effectively utilize computer resources, handle software installations and updates, and troubleshoot common technical issues. The nursing informatics operation skills describes the specific competencies and proficiencies required to effectively use nursing informatics tools, systems, and software applications in daily clinical workflows. And the nursing informatics management skills encompasses the knowledge and skills needed to oversee, coordinate, and optimize the use of nursing informatics in healthcare settings. All items are scored on a 5-point Likert scale from “strongly disagree” to “strongly agree”. Total scores on the scale range from 32 to 160, with higher scores indicating higher levels of NI competency. The NICS demonstrated good reliability and validity, as evidenced by a content validity index of 0.957 and a Cronbach alpha coefficient of 0.947 [24]. In our study, Cronbach’s alpha was 0.979, indicating high internal consistency.

#### Innovative self-efficacy

Innovative self-efficacy was evaluated using the Innovative Self-Efficacy Scale (ISES) [25], adapted and revised by Gu et al. in 2009 [26]. Chinese version of ISES (C-ISES) consists of 8 items that provide responses on a 5-point Likert scale ranging from “strong disagreement” to “strong agreement”. Total scores on the scale range from 8 to 40, with higher scores indicating elevated levels of innovative self-efficacy. Previous studies identified that C-ISES had good reliability, yielding a Cronbach alpha coefficient of 0.886 [26]. The construct validity was analyzed by the exploratory factor analysis (EFA) and confirmatory factor analysis(CFA), the results of EFA showed that the value of KMO of C-ISES was 0.883, cumulative variance contribution rate was 56.3%; CFA verified that C-ISES had a satisfactory model fit with *χ*^*2*^*/df* = 2.861, root-mean-square error of approximation (RMSEA) was 0.073, comparative fit index (CFI) was 0.997 [26]. In our study, Cronbach’s alpha was found to be 0.967.

### Statistical analysis

SPSS 26.0 was used for data analysis in our study. Categorical data were described by calculating frequencies and percentages, while continuous data were presented as the mean and standard deviation (SD). Normality analysis was conducted using the Kolmogorov-Smirnov test, which indicated that none of the variables followed a normal distribution in this study. Therefore, we used the Mann-Whitney *U* test and the Kruskal-Wallis *H* test in a non-parametric test to initially analyze the influencing factors, and the correlation between NI competency and innovation self-efficacy was analyzed using Spearman’s two-sided test, which was a nonparametric measure of rank correlation (statistical dependence between the rankings of two variables). Multivariate linear regression was performed to explore the factors associated with NI competency. The result was considered statistically significant when the two-tailed *p*-value was less than 0.05.

## Results

### Participant characteristics

In this study, the majority of the palliative care nurses were female (98.5%), with 385 (94.1%) holding a bachelor’s degree or higher. Additionally, 239 nurses (53.1%) had worked in palliative care for over 2 years. Details were presented in Table 2.

**Table 2:** Demographic characteristics and univariate analysis of NI competency in palliative care nurses (*n* = 409)

Sociodemographic variables	n (%)	NI competency	Z/H	P
		M(SD)		
**Gender**				
Female	403(98.5)	128.99 ± 1.10	-1.681	0.093
Male	6(1.5)	142.50 ± 5.097		
**Age**				
20-30year	89(21.8)	127.21 ± 2.59	0.794	0.692
31-40year	263(64.3)	130.11 ± 1.32		
≥ 41year	57(13.9)	128.05 ± 2.80		
**Marital status**				
Unmarried	61(14.9)	134.25 ± 2.16	-1.675	0.094
Married	348(85.1)	128.30 ± 1.22		
**Educational background**				
Junior college and below	24(5.9)	122.67 ± 4.47	10.475	0.005*
Bachelor’s degree	366(89.5)	128.93 ± 1.16		
Master’s degree and above	19(4.6)	142.53 ± 2.64		
**Hospital level**				
Tertiary hospitals	356(87.0)	130.65 ± 1.16	14.901	0.001*
Secondary hospitals	38(9.3)	119.87 ± 3.48		
Primary hospitals	15(3.7)	118.27 ± 5.13		
**Professional title**				
Nurse	12(2.9)	133.50 ± 5.31	4.144	0.246
Senior nurse	159(38.9)	125.96 ± 1.99		
Supervisor nurse	206(50.4)	131.21 ± 1.39		
Deputy chief nurse and above	32(7.8)	130.63 ± 3.30		
**Monthly income (RMB)**				
≤ 5,000	80 (19.5)	125.48 ± 2.47	5.203	0.074
5,001–10,000	242(59.2)	129.63 ± 1.34		
≥ 10,000	87(21.3)	131.39 ± 2.66		
**Employment category**				
Contract employee	241(58.9)	128.63 ± 1.52	0.196	0.907
Personnel agency employee	38(9.3)	129.18 ± 2.96		
Formal employee	130(31.8)	130.23 ± 1.76		
**Years of working experience in palliative care**				
≤ 1year	170(41.6)	127.86 ± 1.64	2.227	0.527
2-5year	143(35.0)	131.38 ± 1.59		
5-10year	58(14.1)	128.71 ± 3.49		
≥ 10year	38(9.3)	127.61 ± 4.45		

*indicates *P* < 0.05

### NI competency

The mean scores of NI competency total and NI awareness, computer operational capability, computer management capability, NI operation skills, NI management skills dimensions were 129.19 ± 22.02, 15.51 ± 2.99, 33.47 ± 6.20, 20.98 ± 3.96, 12.77 ± 2.50, 46.46 ± 9.21, respectively, suggesting that Chinese palliative care nurses in this study generally had moderate levels of NI competency [27]. The item average score of NI operation skills dimension had the highest score at 4.26 ± 0.83, which means that most palliative care nurses had a high level of proficiency in the operation of nursing information systems. The NI management skills dimension had the lowest score at 3.87 ± 0.77, which means that the nurses lacked the related knowledge and skills needed to oversee, coordinate, and optimize the use of nursing informatics in healthcare settings. The total score of the C-ISES please see our previous work [28] (3).

**Table 3:** Mean scores of NICS and C-ISES (*n* = 409)

	Numbers of items	Score M(SD)	Item average score M(SD)
NICS total	32	129.19(22.02)	4.03(0.69)
NI awareness	4	15.51(2.99)	3.88(0.75)
Computer operational capability	8	33.47(6.20)	4.18(0.78)
Computer management capability	5	20.98(3.96)	4.20(0.79)
NI operation skills	3	12.77(2.50)	4.26(0.83)
NI management skills	12	46.46(9.21)	3.87(0.77)

### Factors related to NI competency

#### Demographic factors influencing NI competency

The results of the univariate analysis were shown in Table 2. Nurses with a bachelor’s or above degree had higher scores than the ones with a Junior college or below educational level (*H* = 10.475, *P* = 0.005). Nurses who worked in secondary-level hospitals and above had higher NI competency than primary-level hospitals (*H* = 14.901, *P* = 0.001).

#### Correlation between the innovative self-efficacy and NI competency

The Spearman correlation indicated a positive association between innovative self-efficacy and NI competency (*r* = 0.602, *P* < 0.01). The dimensions of NI awareness (*r* = 0.543, *P* < 0.01), computer operational capability (*r* = 0.472, *P* < 0.01), computer management capability (*r* = 0.432, *P* < 0.01), NI operation skills (*r* = 0.396, *P* < 0.01), NI management skills (*r* = 0.606, *P* < 0.01) were also positively correlated with innovative self-efficacy, respectively, as highlighted in Table 4.

**Table 4:** Results of Spearman correlations analysis

	NI competency	NI awareness	Computer operational capability	Computer management capability	NI operation skills	NI management skills	Innovative self-efficacy
**NI competency**	1.000	-	-	-	-	-	-
NI awareness	0.716^**^	1.000	-	-	-	-	-
Computer operational capability	0.862^**^	0.592^**^	1.000	-	-	-	-
Computer management capability	0.860^**^	0.536^**^	0.778^**^	1.000	-	-	-
NI operation skills	0.839**	0.508**	0.728**	0.842**	1.000	-	-
NI management skills	0.912**	0.594**	0.665**	0.694**	0.708**	1.000	-
**Innovative self-** **efficacy**	0.602**	0.543**	0.472**	0.432**	0.396**	0.606**	1.000

**indicates *P* < 0.01

#### Multiple linear regression analysis of NI competency

A multiple linear regression analysis was conducted to the explore the factors associated with nurses’ NI competency, using NI competency as the dependent variable and the statistically significant variables identified in Tables 2 and 4 as the independent variables. The specific assignment for independent variables were shown in Table 5. The linear regression equations demonstrated a favorable fit to the data (*F* = 74.818, *P* < 0.001), which explained 35.2% of the variances. The innovative self-efficacy (*β* = 0.567, *P* <0.001) and hospital level (*β* = -0.103, *P* < 0.012) emerged as significant factors influencing NI competency, as highlighted in Table 6.

**Table 5:** Assignment of independent variables

Variables	Assignment method
Educational background	1 = Junior college and below, 2 = Bachelor’s degree, 3 = Master’s degree and above
Hospital level	1 = Tertiary hospitals, 2 = Secondary hospitals, 3 = Primary hospitals
Innovative self-efficacy	Measure value

**Table 6:** Multiple linear regression analysis of NI competency

Variables	B	SE	β	t	P
Constant	63.769	7.422	-	8.591	< 0.001**
Innovative self-efficacy	2.159	0.153	0.567	14.084	< 0.001**
Hospital level	-5.004	1.927	-0.105	-2.597	0.010*
Educational background	4.052	2.745	0.060	1.476	0.141

*R* = 0.597, *R*^2^ = 0.357, Adjusted *R*^2^ = 0.352, *F* = 74.818, *P* < 0.001

*indicates *P* < 0.05, **indicates *P* < 0.001

## Discussion

As palliative care services become increasingly technology-driven worldwide, the significance of developing NI competency becomes even more pronounced, as it is essential for delivering efficient and patient-centred continuity of care. To our best of knowledge, the present study is the first study to investigate the current situation of NI competency among palliative care clinical nurses in mainland China, which provides a new valuable insight into this field. The total score of NICS was 129.19 ± 22.02, which indicated that Chinese palliative care nurses had a moderate level of NI competency. The hospital level and innovative self-efficacy were identified as statistically significant factors influencing nurses’ NI competency based on multiple linear regression analysis results.

The findings of the current study indicate that the dimension of NI operational skills within NI competency exhibits the highest average scores for the items, indicating that nurses have a high level of proficiency in the operation of nursing information systems. The result was also supported by a study conducted by Zhu et al. [27]. In recent years, with the rapid development of Internet medical services, most Chinese nursing schools have actively introduced nursing informatics courses that encompass both theoretical and practical aspects, which can contribute to improving the NI operation skills of nurses to a certain extent [29]. However, the dimension of NI management skills shows the lowest average score for items, similar to the findings of He et al. [30]. Despite the growing recognition of the importance of nursing informatics in healthcare, there may be gaps in the education and training provided to nurses regarding the management of information systems. In China, most nursing informatics courses tend to focus more on the practical operation of nursing information systems, leading to a relative lack of emphasis on essential skills related to effective management, such as nursing information integration and nursing information security management [21]. As a result, most nurses may exhibit a certain level of deficiency in confidence and proficiency in these critical areas. Moreover, the complexity of the healthcare environment and the numerous clinical tasks and responsibilities shouldered by palliative care nurses may also contribute to the lower item average scores for management skills in NI. The workload of palliative care nurses is further intensified when attending to patients in the end-stage of their disease, as these individuals frequently exhibit unstable conditions [31], leaving them with limited time and resources to devote to managing information systems.

In 2022, a similar study was conducted by Chen et al. examining NI competency among 380 nurses specializing in inflammatory bowel disease [32]. The study reported a NI competency score of 122.79 ± 22.53, slightly lower than that observed among palliative care nurses. In addition to variations in the nurse survey population, the disparity in results can be attributed to the divergence in patient populations between the two specialties. Palliative care nurses often encounter a diverse patient population with unique care needs, particularly for terminal cancer patients receiving home-based palliative care, most of whom require remote symptom management, psychological intervention, and end-of-life daily care [33], requiring a higher level of informatics competency to effectively manage and coordinate care. On the other hand, nurses specializing in inflammatory bowel disease may have a more focused patient population with a narrower range of interventions, leading to a relatively lower level of NI competency. Moreover, as Chinese government policies continue to advance, the implementation of DH-based palliative care services has demonstrated tangible enhancements in clinical practice, notably enhancing nurse efficiency [11]. The positive impact has fostered an increased willingness among most nurses to actively engage in the delivery of DH-based palliative care services, which may explain why palliative care nurses have a higher level of informatics competency than other specialist nurses.

In our study, we found there was a notable association between nurses’ hospital levels and their NI competency levels. Specifically, nurses in higher-level hospitals demonstrated higher NI competency levels than those in lower-level hospitals. One possible reason for this trend is that higher-level hospitals tend to place a greater emphasis on investment in medical information construction [27]. In China, the robust healthcare information infrastructure in tertiary hospitals enables nurses to efficiently navigate through DH tools, access up-to-date patient data, and implement evidence-based care practices. As a result, nurses in these higher-level hospitals have more opportunities to engage with and apply nursing information systems in their day-to-day work, thereby facilitating the development of their NI competency. However, for most primary-level hospitals in mainland China, their capacity is limited to delivering basic public health services, and the allocation of medical information resources and education and training opportunities for medical personnel is relatively inadequate [34]. As a result, nurses in these settings may have limited exposure to care information systems, resulting in fewer opportunities to enhance their NI competency. To further improve the NI competency of nurses in primary-level hospitals, the nursing education managers of primary-level hospitals can develop supported strategies such as targeted informatics training programs, improved access to medical information resources and collaborate with higher-level healthcare institutions to prompt nurses to carry out a comprehensive and in-depth understanding of knowledge and skills related to NI [35]. Moreover, future studies can focus more on comprehensively evaluate the specific barriers and challenges faced by nurses in primary hospitals related to limited exposure to NI systems through qualitative methods, so as to provide valuable knowledge for the development of targeted measures.

In addition, our study found a statistically significant correlation between innovative self-efficacy and NI informatics competency among palliative care nurses, suggesting that nurses with higher levels of innovative self-efficacy are more likely to exhibit more significant levels of NI competency. In the context of palliative care, where nurses are confronted with the complex care needs of end-of-life patients, those with a higher level of innovative self-efficacy tend to be more inclined to explore and embrace new technologies or interventions that have the potential to enhance patient care and improve outcomes [36]. Moreover, nurses with innovative self-efficacy are more proactive in seeking learning and professional development opportunities in informatics. They are motivated to stay up-to-date with the latest advancements in technology and informatics practices, allowing them to improve their NI competency continuously [19, 37]. To maximize the benefits of innovative self-efficacy in improving NI competency, healthcare institutions can foster a favorable climate for innovation and offer a diverse range of innovative activities while emphasizing the provision of targeted and specialized informatics training for palliative care nurses.

## Limitations

First, this study used a convenient sample and just recruitment participants in mainland China. Therefore, the results may not be entirely representative of the NI competency landscape among palliative care nurses in different cultural or geographical contexts, potentially impacting the generalizability of the findings. Future research endeavors are encouraged to further improve the representativeness of sample size and account for diversities in cultural and geographical contexts when investigating NI competency among palliative care nurses, which would facilitate a more comprehensive understanding of NI competency within varying cultural frameworks. Second, given the ongoing technological advancements in healthcare informatics systems, nurses need to continually adapt and acquire new skills to utilize these DH-based tools effectively in their practice. Therefore, the NI competency among nurses may be changed over time. Future research could employ a longitudinal study design to comprehensively explore the trajectory of informatics competency among palliative care nurses over an extended period. Finally, given that healthcare systems can significantly differ across regions and countries, comparing the findings of this study with those in different healthcare settings may complicate the interpretation of NI competency scores. To facilitate more accurate interpretations and comparisons of NI competency among palliative care nurses, it is recommended that researchers delve deeper into investigating the potential interplay between healthcare system variances and NI competency to offer valuable insights to the field.

## Conclusions

This study found that palliative care nurses in mainland China exhibited moderate levels of NI competency and identified the hospital level and innovative self-efficacy of nurses were significant factors influencing NI competency, with nurses in higher-level hospitals and with higher innovative self-efficacy displaying higher NI competency levels than those in lower-level hospitals and with lower innovative self-efficacy. The findings of this study help nurse managers better understand NI competency among palliative care nurses in mainland China. Measures such as developing supported strategies, including targeted NI training programs by nursing education managers of primary-level hospitals and creating a positive culture of innovation by healthcare institutions can be considered to improve the level of NI competency among Chinese palliative care nurses.

### Electronic supplementary material

Below is the link to the electronic supplementary material.

Supplementary Material 1

Supplementary Material 2

Supplementary Material 3

## Data Availability

The data used during this study are available from the corresponding authors on reasonable request.
