# Supplementary material for: Nursing informatics competency and its associated factors among palliative care nurses: an online survey in mainland China
Source: BMC Nurs. 2024 Mar 5;23:157. doi: 10.1186/s12912-024-01803-5 (PMC10913251; doi:10.1186/s12912-024-01803-5)
Supplement: Supplementary file 1 — Supplementary Material 1 [file 12912_2024_1803_MOESM1_ESM.docx]

**Appendix** **II Nursing Informatics Competency Scale (NICS)**

1.In nursing work or research, I have a good sensitivity to information

□Strongly disagree

□Disagree

□Neutral

□Agree

□Strongly agree

2.I am able to express informatics needs in detail and identify multiple forms of informatics sources.

□Strongly disagree

□Disagree

□Neutral

□Agree

□Strongly agree

3.I have the awareness to cooperate with other medical personnel to obtain, process, and exchange information.

□Strongly disagree

□Disagree

□Neutral

□Agree

□Strongly agree

4.I am an active advocate for the application of informatics concepts to the nursing profession.

□Strongly disagree

□Disagree

□Neutral

□Agree

□Strongly agree

5.I am able to master word processing skills, such as Microsoft WORD documents.

□Strongly disagree

□Disagree

□Neutral

□Agree

□Strongly agree

6.I am able to master the skills of table processing, such as Microsoft EXCEL tables.

□Strongly disagree

□Disagree

□Neutral

□Agree

□Strongly agree

7.I am able to master presentation skills, such as Microsoft Power Point.

□Strongly disagree

□Disagree

□Neutral

□Agree

□Strongly agree

8.I am able to perform basic computer operations (e.g., power on, power off, paper loading, handling paper clips).

□Strongly disagree

□Disagree

□Neutral

□Agree

□Strongly agree

9.I have good typing skills.

□Strongly disagree

□Disagree

□Neutral

□Agree

□Strongly agree

10.can find and download important information online (e.g., patient and nursing information).

□Strongly disagree

□Disagree

□Neutral

□Agree

□Strongly agree

11.I am proficient in email related functions (writing, sending, replying to emails, etc.).

□Strongly disagree

□Disagree

□Neutral

□Agree

□Strongly agree

12.In nursing work, I am skilled in effectively connecting computer monitoring systems with patients.

□Strongly disagree

□Disagree

□Neutral

□Agree

□Strongly agree

13.I can monitor the patient's condition with a computerized monitoring system.

□Strongly disagree

□Disagree

□Neutral

□Agree

□Strongly agree

14.I can use computer software to process patient care files.

□Strongly disagree

□Disagree

□Neutral

□Agree

□Strongly agree

15.I can develop a care plan using computer software.

□Strongly disagree

□Disagree

□Neutral

□Agree

□Strongly agree

16.I can utilize the application to document the patient's care process.

□Strongly disagree

□Disagree

□Neutral

□Agree

□Strongly agree

17.I am able to use the application to enter relevant information about the patient (vital signs and physiological parameters, etc.).

□Strongly disagree

□Disagree

□Neutral

□Agree

□Strongly agree

18.I am proficient in the use of automatic identification techniques and equipment in nursing work (e.g., using a "bar code scanner" to identify patient bar codes).

□Strongly disagree

□Disagree

□Neutral

□Agree

□Strongly agree

19.I am proficient in the use of communication technology and equipment (e.g., patient paging systems) to facilitate effective communication in a variety of care settings.

□Strongly disagree

□Disagree

□Neutral

□Agree

□Strongly agree

20.I am proficient in administering medications utilizing an inpatient medication management system.

□Strongly disagree

□Disagree

□Neutral

□Agree

□Strongly agree

21.I am able to identify problems in the nursing informatics process/nursing informatics system in a timely manner.

□Strongly disagree

□Disagree

□Neutral

□Agree

□Strongly agree

22.I am able to participate in the selection, design, implementation, evaluation and maintenance of nursing informatics systems.

□Strongly disagree

□Disagree

□Neutral

□Agree

□Strongly agree

23.I can make nursing diagnoses based on standardized nursing language systems such as the NANDA diagnostic classification system.

□Strongly disagree

□Disagree

□Neutral

□Agree

□Strongly agree

24.I have the ability to save and integrate nursing information.

□Strongly disagree

□Disagree

□Neutral

□Agree

□Strongly agree

25.I am able to identify, evaluate and use care-related information.

□Strongly disagree

□Disagree

□Neutral

□Agree

□Strongly agree

26.I can use data and information to analyze, evaluate, and describe clinical nursing practices.

□Strongly disagree

□Disagree

□Neutral

□Agree

□Strongly agree

27.I can use data, information, and knowledge critically.

□Strongly disagree

□Disagree

□Neutral

□Agree

□Strongly agree

28.I have the ability to turn data into information, knowledge and wisdom.

□Strongly disagree

□Disagree

□Neutral

□Agree

□Strongly agree

29.I am able to use the information gained to develop a nursing research plan and write a research paper.

□Strongly disagree

□Disagree

□Neutral

□Agree

□Strongly agree

30.I am proficient in the use of nursing informatics systems, informatics technology and communication equipment for nursing safety management.

□Strongly disagree

□Disagree

□Neutral

□Agree

□Strongly agree

31.I am proficient in utilizing nursing informatics systems for nursing manpower management.

□Strongly disagree

□Disagree

□Neutral

□Agree

□Strongly agree

32.I am skilled in utilizing nursing informatics systems, informatics technology to communicate effectively in the nursing process.

□Strongly disagree

□Disagree

□Neutral

□Agree

□Strongly agree
