# Supplementary material for: Nursing informatics competency and its associated factors among palliative care nurses: an online survey in mainland China
Source: BMC Nurs. 2024 Mar 5;23:157. doi: 10.1186/s12912-024-01803-5 (PMC10913251; doi:10.1186/s12912-024-01803-5)
Supplement: Supplementary file 2 — Supplementary Material 2 [file 12912_2024_1803_MOESM2_ESM.docx]

**Appendix I Demographic Information Questionnaire**

1.What is your gender？

□Male □Female

2.What is your age?

□20-30year □31-40year □41-50year □≥50year

3.What is your marital status?

□Unmarried □Married □Divorced □Widowed

4.What is your educational background?

□Junior college and below □Bachelor’s degree □Master’s degree

□Ph.D. degree and above

5.What is your hospital level?

□Tertiary hospitals □Secondary hospitals □Primary hospitals

6.What is your professional title?

□Nurse □Senior nurse □Supervisor nurse □Deputy chief nurse and above

7.What's your monthly income(RMB)?

□≤3,000 □3,000-5,000 □5,000-10,000 □≥10,000

8.What's your employment category?

□Contract employee □Personnel agency employee □Formal employee

□Temporary employee

9.How long have you worked in palliative care?

□≤1year □2-5year □5-10year □≥10year
