# Supplementary material for: Nursing informatics competency and its associated factors among palliative care nurses: an online survey in mainland China
Source: BMC Nurs. 2024 Mar 5;23:157. doi: 10.1186/s12912-024-01803-5 (PMC10913251; doi:10.1186/s12912-024-01803-5)
Supplement: Supplementary file 3 — Supplementary Material 3 [file 12912_2024_1803_MOESM3_ESM.docx]

**Appendix III Chinese version of the Innovative Self-Efficacy Scale (ISES)**

1.I can accomplish the majority of my goals through innovative ways of working.

□Strong disagreement

□Disagreement

□Neutral

□Agreement

□Strong agreement

2.Faced with difficult tasks, I'm pretty sure I'll be able to do it creatively.

□Strong disagreement

□Disagreement

□Neutral

□Agreement

□Strong agreement

3.Overall, I feel that I can innovatively make significant achievements.

□Strong disagreement

□Disagreement

□Neutral

□Agreement

□Strong agreement

4.Most of the time, I can turn an innovative idea into a reality.

□Strong disagreement

□Disagreement

□Neutral

□Agreement

□Strong agreement

5.I can deal with all kinds of challenges creatively.

□Strong disagreement

□Disagreement

□Neutral

□Agreement

□Strong agreement

6.I am confident in my creative ability to accomplish various tasks.

□Strong disagreement

□Disagreement

□Neutral

□Agreement

□Strong agreement

7.Compared to others, I am very creative in my work.

□Strong disagreement

□Disagreement

□Neutral

□Agreement

□Strong agreement

8.Even when things are difficult, I still do it creatively.

□Strong disagreement

□Disagreement

□Neutral

□Agreement

□Strong agreement
